# Metal Ion-/Proton-Coupled Electron Transfer (MPCET) on *ortho*-Quinone

**DOI:** 10.1021/acsomega.4c03621

**Published:** 2024-09-05

**Authors:** Divyaratan Kumar, Viktor Gueskine, Ziyauddin Khan, Reverant Crispin, Mikhail Vagin

**Affiliations:** † Laboratory of Organic Electronics, Department of Science and Technology, Linköping University, Norrköping SE-60174, Sweden; ‡ Wallenberg Wood Science Center, ITN, Linköping University, Norrköping SE-60174, Sweden; § Wallenberg Initiative Materials Science for Sustainability, Department of Science and Technology, Linköping University, Norrköping SE-60174, Sweden

## Abstract

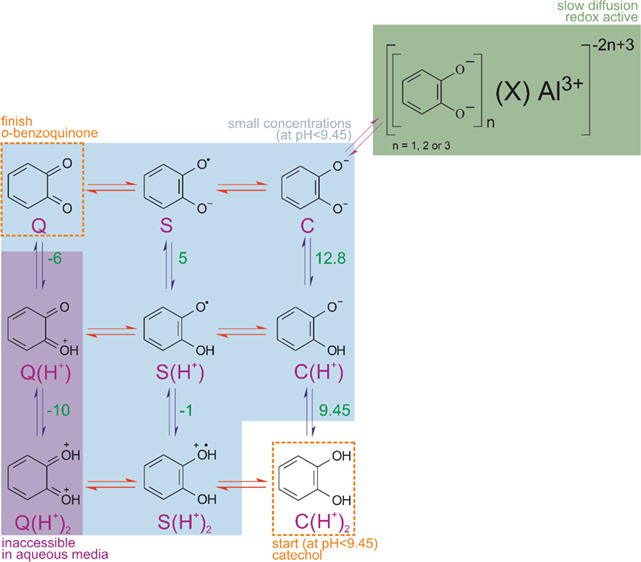

Quinol/quinone equilibria are ubiquitous in nature and find multiple technological applications, most recently in electrical charge storage. Much research has been devoted to proton-coupled electron transfer (PCET) in such systems and to bidentate complexation of *ortho*-quinol (catechol) ligands with multivalent metal ions but rarely to the interplay of these two reactions. Here, we investigate the impact of a redox-inactive metal ion, as a complexing and charge-compensating agent, on redox processes of catechol in aqueous solutions, that is, in the presence of proton equilibria. We pay separate attention to their thermodynamics and kinetics, which can be regulated by the pH and buffer capacity. As the proton buffer concentration decreases, proton equilibria during catechol PCET are slower to establish, thus kinetically prioritizing the participation of the metal ion rather than the proton in the redox charge compensation. Making use of this kinetic interplay can be a general strategy to conceive organic battery cathodes for proton-free metal-ion aqueous batteries.

## 1 Introduction

The fascinating chemistry of quinol/quinone (e.g., catechol as quinol and o-benzoquinone as its quinone counterpart) includes, on the one hand, the two-electron redox process of this couple and, on the other hand, the ability of quinols to form complexes with metal ions. Complexation can be particularly strong with catechols, where the ortho-position of the hydroxyls leads to chelation when the metal ion is prone to multiple coordination.

Noteworthily, redox and complexation are both related to acid–base equilibria on the quinol/quinone couple. All three processes are reversible, creating intriguing complexity in this system. Nature makes wide use of all of these functions. Multistep proton-coupled electron-transfer (PCET) chains involving quinones as key molecules define the abundance of such molecular systems in vivo: the photosystem II,^1^ ADP-to-ATP oxidative phosphorylation in the mitochondria,^2^ and neurochemistry.^3^*o*-Quinones (often designating quinols and quinones together) are among the most explored organic redox systems.^4−8^ The natural ability of many marine organisms to attach themselves to underwater surfaces, such as the amazing adhesion of mussel foot proteins, is based on the catechol functionality^9^ to form bonds with the surface primarily via the bidentate in vivo complexation of catechol-modified proteins with transition metal ions, as confirmed by the accumulation of these metal species in the adhesives of mussels with respect to their levels in the marine environment.^10^ Humans have mimicked nature since the Middle Ages in utilizing Fe^2+/3+^ chelation by polyphenols to prepare gall ink^11^ and, more recently, in a variety of functional coatings with different metal ions.^12^*o*-Dioxolene complexes may exhibit valence tautomerism^13^ if the metal ion can also exist in various oxidation states, which may be relevant for in vivo bioadhesion^14^ and spintronics.^15^

The high density of electrical charge determined by the reversible redox process involving two electrons per elementary quinone unit (e.g., catechol) and their natural abundance inspired the idea of using quinones for electrical energy storage.^16^ Metal ions are the only species involved in ionic processes in secondary metal-ion batteries constructed with quinones as cathode materials and based on aprotic electrolytes. When quinones are used in aqueous batteries,^17^ the conversive redox process upon discharge was supposed to lead to the formation of coordination complexes between the metal ions and reduced catechol.^18^ The competition between the metal ion complexation and PCET on the redox process of quinones was most often optimistically ignored. The situation becomes even more complex on films limited by mass transport. Only recently, the effect of the selectivity of redox processes toward MCET, and not toward PCET, in nominal metal-ion insertion cathodes on the battery performance was recognized.^19^

In this work, we address the basis of such phenomena: the redox behavior of catechol in solution considering competitive interactions with metal ions and protons. We conceptualize this as a metal ion-/proton-coupled electron transfer (MPCET). We selected aluminum ions for this study due to their ability to efficiently associate with catechol^20−22^ combined with the absence of redox activity. We used different buffer systems as well as experimental and theoretical tools to decouple MCET and PCET in the catechol redox process.

## 2 Experimental Section

### 2.1 Chemicals and Materials

The 4-(2-hydroxyethyl)piperazine-1-ethanesulfonic acid (HEPES) buffer mixture was prepared using *N*-(2-hydroxyethyl)piperazine-*N*′-(2-ethanesulfonic acid), 1 M HEPES, 0.05 M potassium chloride (KCl), 1 mM 1,2-dihydroxybenzene catechol, and 0.5 mM aluminum chloride (AlCl_3_). All solutions were prepared in DI water.

Borate-free Britton–Robinson buffers of different pH values were prepared using sodium hydroxide (NaOH), acetic acid (CH_3_COOH), phosphoric acid (H_3_PO_4_), and potassium chloride (KCl) in different ratios (Table S1).

### 2.2 Electrochemical Experiment

All electrochemical experiments were performed with a BioLogic SP 200 potentiostat three-electrode electrochemical cell using a platinum wire as the auxiliary electrode and Ag/AgCl (3 m KCl) as the reference electrode in aqueous media. A glassy carbon electrode (GCE, 5 mm diameter) was utilized as a working electrode. Prior to use, GCE was successively polished with pk31.0 and 0.05 μm Al_2_O_3_ alumina pads and sonicated in Milli-Q water. The rotating disk ring electrode setup (5 mm OD GCE, 320 μm gap, platinum ring 6.25 mm ID, 7.92 mm OD; Pine Research Instrumentation Inc.) was utilized for the control of the rotation speed. The pH was continuously measured using a glass Thermo Scientific OrionStar Bench pH Meter Kit with 5 points calibrated.

### 2.3 Theoretical Calculations

The equilibrium concentration of the species was calculated with the help of the free software ChemEQL V.3.2^23^ using the complexation and dissociation constants available in the ChemEQL database completed by the data from the literature.^22^ Simulated cyclic voltammograms were obtained using DigiSim 3.0 software (BASi Inc., West Lafayette, IN, USA).

## 3 Results and Discussion

### 3.1 Background

**Scheme 1:**
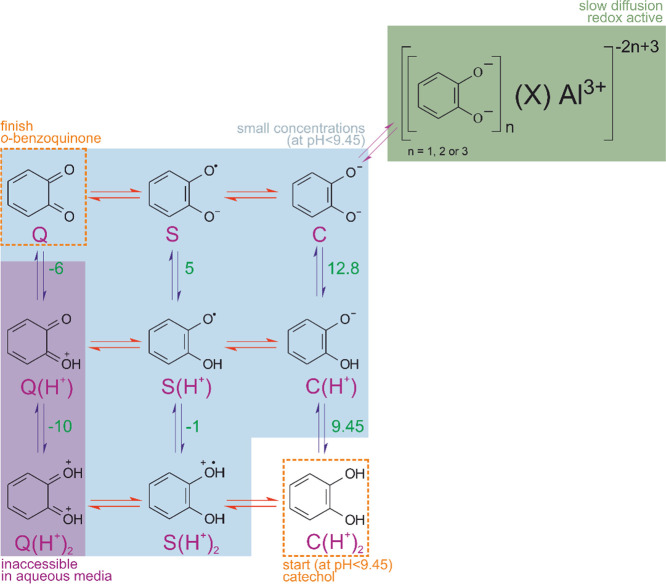
Scheme of Squares of Catechol Oxidation pKa values for catechol from refs (4, 6, 7, 24, and 30); red (horizontal) and blue (vertical) arrows represent electron transfers and proton equilibria, respectively; diagonal arrows represent the aluminum complexation. Reprinted (adapted or reprinted in part) with permission from ref (7). Copyright 2014 American Chemical Society.

Following the notation by Compton’s group,^7^ we designate the deprotonated redox states of the quinol/quinone redox couple (Scheme 1; quinone, semiquinone, and catechol denoted as *Q*, *S*, and *C*, respectively) and explicitly indicate the number of protons bound to phenolic oxygen atoms.

The overall PCET of quinones in protic solvents including water can then be represented as follows:
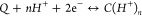
1where *n*, the number of protons involved as a Bro̷nsted acid, can be 0, 1, or 2 depending on the pH. All possible intermediates can be assembled in a nine-membered Scheme of Squares (Scheme 1),^4,7,8,24−26^ where horizontal arrows represent the electron transfer between the three redox states (*Q*, *S*, and *C*) and vertical arrows represent elementary steps involving protons.

The overall MCET with a redox-inactive metal ion on quinones can be stoichiometrically represented as follows: 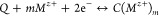
2where for *z* > 1, *m*, the number of metal ions involved as a Lewis acid replacing protons in eq 1, can be fractional if more than one catechol is bound to one ion. An aluminum ion can bind one, two, or three catechols, giving *m* = 1, , or , respectively. The complexation with *Q* can be neglected due to the much higher complexation ability between anions of catechol, namely *C* and *C*(*H*^+^), and metal cations.

In aqueous media, where protons and metal cations coexist, the selectivity between PCET and MCET should depend on the competition between protonation and metal complexation.

Inclusion of MCET (reaction 2) in parallel with PCET (reaction 1) requires an upgrade of the Scheme of Squares (Scheme 1) by adding new equilibria. The interim character of the PCET intermediates implies that their concentrations available for complexation are negligible. Although a general background electrolyte cation can contribute to MCET, a multivalent metal ion should offer a thermodynamic merit: complexation yielding a variety of catechol complexes of high association constants.^21,22^

A famous analytical model for PCET was built by Laviron, further assuming the rate of the proton-associated steps to be infinitely high with respect to the electron-transfer rate, which assures the establishment of the proton equilibria at every potential at the cyclic voltammetry time scale.^26^ This assumption of the fast establishment of proton equilibria during the quinone redox process relies on a *high proton buffer capacity* of the media. The pH of the aqueous media with respect to the relevant acidity constants of *Q* and *S* then determines the number of protons involved in reaction 1. Noteworthily, the effect of multivalent metal ions, e.g., aluminum or zinc species, on the pH in the media of *low proton buffer capacity* is double: such ions are prone to hydrolysis and also take part in proton equilibria by binding to catechol and liberating protons, thus jeopardizing the decoupling of MCET from PCET. Therefore, to mitigate these complications in our study of PMCET, we focused on well-buffered systems first.

#### 3.1.1 Catechol–Aluminum in Britton–Robinson Buffer

**Figure 1:**
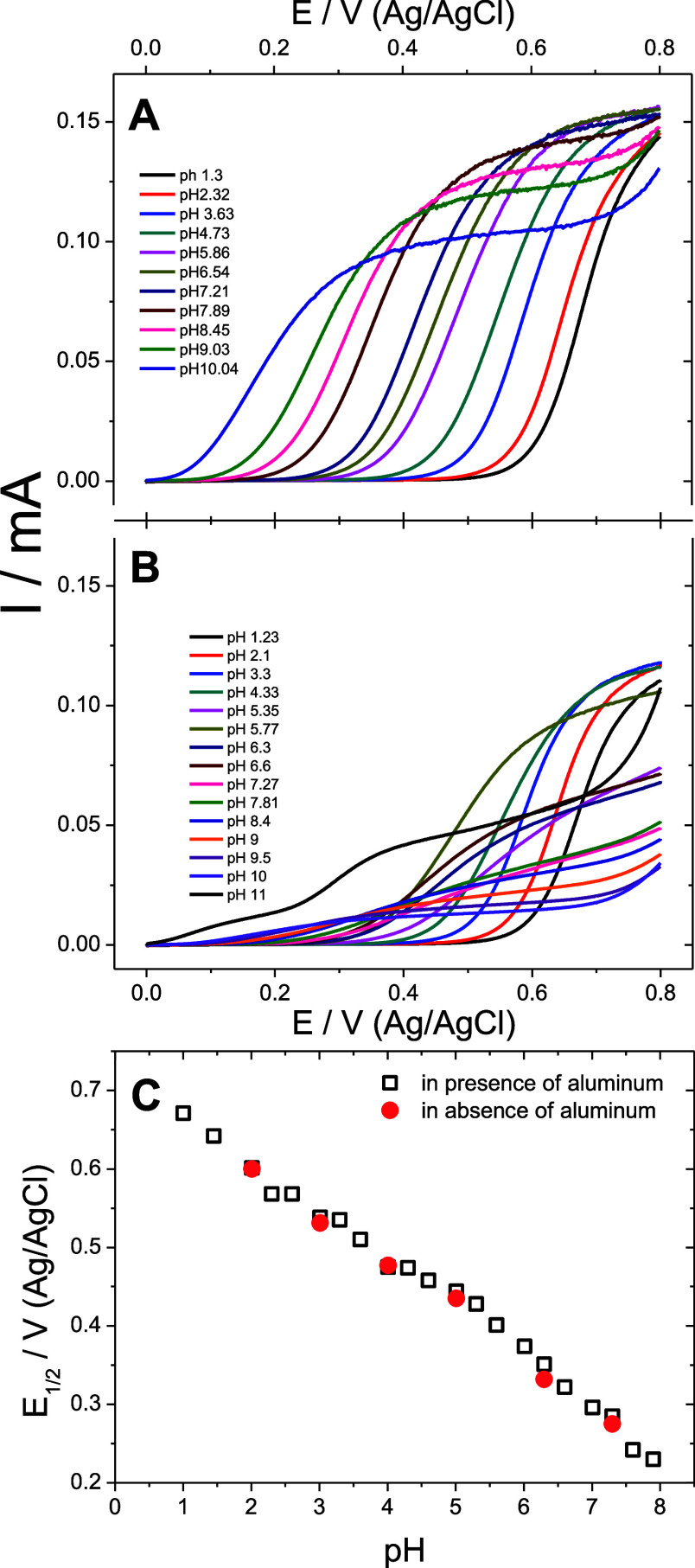
Intactness of the catechol oxidation thermodynamics in the presence of aluminum species in buffered aqueous electrolytes. Linear sweep voltammograms obtained on glassy carbon RDE (rotation rate 2500 rpm) in a solution of catechol (0.02 mM) in the Britton–Robinson buffer in the (A) absence and (B) presence of aluminum(III) species (in form of 0.1 mM AlCl_3_; scan rate 20 mVs^–1^). (C) Pourbaix diagrams of the half-wave potential of catechol oxidation in the absence and presence of a 5 times excess of aluminum(III) in the Britton–Robinson buffer.

Our goal here is to monitor the redox process in an aqueous catechol-containing electrolyte solution as a voltammetric probe of pH-dependent complexation of catechol assuming that aluminum complexes of catechol would manifest somehow in cyclic voltammetry. The family of borate-free Britton–Robinson buffers (Table S1) was used to control the pH over a wide range while maintaining similarity in composition. The exclusion of borate was motivated by the formation of a complex between catechol and boronic acid.^27^ Aluminum complexation with catechol should be favored to make it readable by voltammetry, so a 5-fold excess of aluminum vs catechol was used. At the same time, to avoid the precipitation of insoluble compounds, the concentrations cannot be too high: 0.02 mM catechol and 0.1 mM aluminum species. However, with a low catechol concentration, the visibility of redox currents in voltammetry is too low with respect to the background (Figure S1). Thus, we utilized a glassy carbon rotating disk electrode (RDE) to observe the catechol oxidation clearly. Due to enhanced convection toward the RDE, higher currents of oxidation were indeed recorded, and a wave-shaped curve (Figure 1A) was observed.

Catechol oxidation manifests as a unified two-electron wave because two elementary electron transfers are merged due to the so-called potential inversion. Specific for quinone redox processes in aqueous media at any pH, the second monoelectronic oxidation (e.g., *S* → *Q*) appears at a much higher driving force than the first monoelectronic oxidation (e.g., *C* → *S*).^7^ The half-wave potential of this bielectronic oxidation shifts toward negative potentials (Figure 1A) with increasing pH as deprotonated forms in the PCET Scheme of Squares are easier to oxidize (upward in Scheme 1). The pH dependence of the half-wave potential of catechol oxidation, the so-called Pourbaix diagram (Figure 1C), showed linearity with a slope of 59 mV per pH unit at pH values below the p*K*_1_ of catechol (9.45). This Nernstian slope indicates that the proton equilibria of catechol oxidation (reaction 1) are established at all pH values of this study on the voltammetry time scale.

In the presence of aluminum species in the borate-free Britton–Robinson buffer, two types of voltammetric responses are observed on the RDE (Figure 1B). At pH values below 5, the curves are similar to those in the absence of aluminum (Figure 1A), while at higher pH values, the currents are significantly lower. However, the half-wave potentials showed the same Nernstian behavior as observed for free catechol (Figure 1C) implying that the catechol oxidation thermodynamics defined by the proton equilibria (reaction 1) remains intact. According to analytical speciation (calculation of the equilibrium concentrations), there are practically no aluminum complexes with catechol species at any pH, as aluminum complexation with the phosphates of the buffer ([*AlH*_2_*PO*_4_]^+^ at pH < 10.5 and [*AlHPO*_4_]^2+^ at pH > 10.5) is more robust (Supporting Note 1). The absence of aluminum effect on the half-wave potentials is thus in coherence with the calculated speciation and underscores the intactness of the Nernstian acid–base equilibria. In contrast, the suppression of redox currents by aluminum must be due to a kinetic nonthermodynamic effect.

#### 3.1.2 Catechol–Aluminum in HEPES Buffer

**Figure 2:**
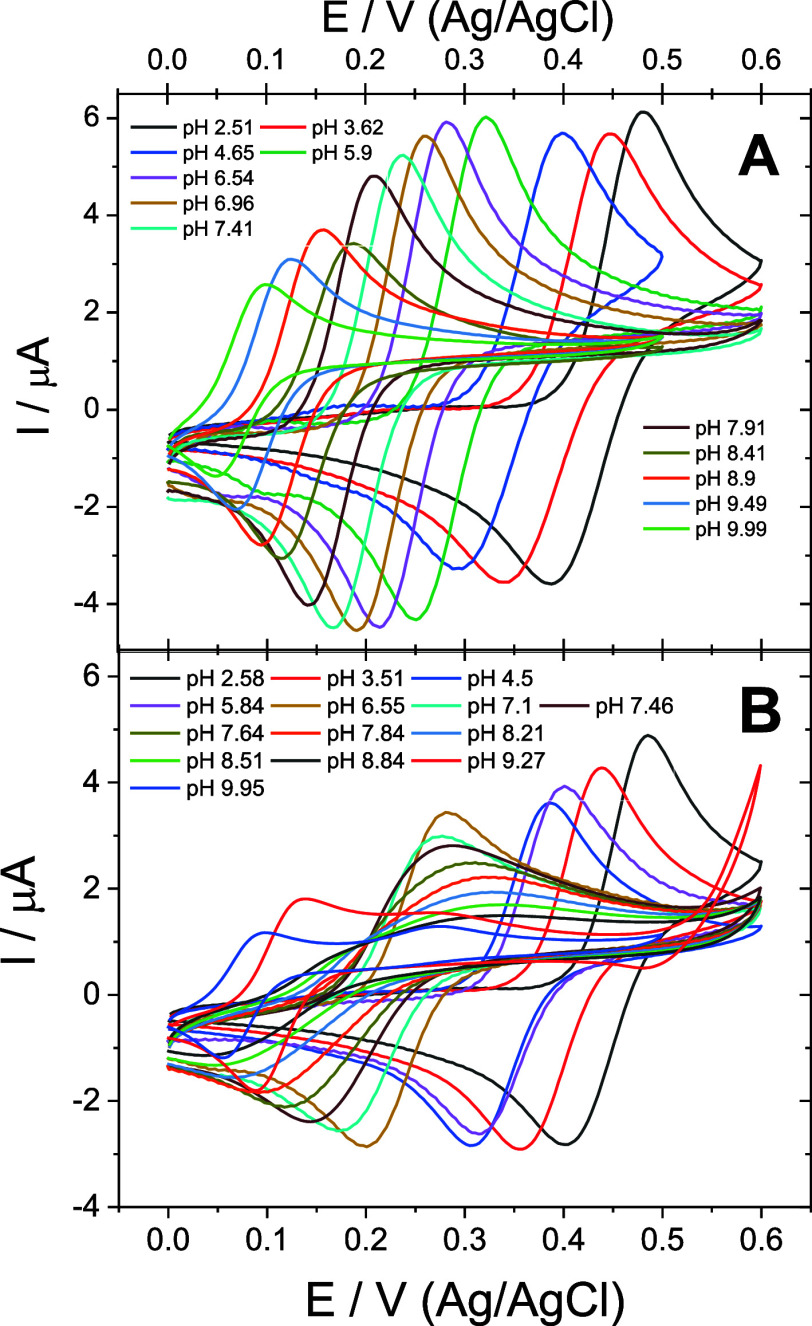
Effect of the aluminum complex formation on the voltammetric response of catechol. Cyclic voltammograms of the catechol redox process (1 mM) recorded on glassy carbon in aqueous HEPES (1 M, 0.05 M KCl) in the absence (A) and presence (B) of aluminum species (0.5 mM salt).

We changed the buffer system to a noncomplexing HEPES buffer from the so-called Good’s buffer family^20^ utilized in biochemistry to keep metal-containing cofactors intact in parallel to buffering. Voltammetry on stagnant electrodes showed a reversible two-electronic redox process of catechol (Figure 2). The effect of the added aluminum on the voltammetric responses (Figure 2A,B, respectively) does not manifest at pH values roughly below 6.5, as both the shape and the position of the voltammetry peak currents remain the same. However, a further increase of pH up to 9 in the presence of aluminum leads to a higher potential difference between the oxidation and reduction peaks, while the peak currents decrease drastically. In other words, the presence of aluminum species led to the decrease of the apparent reversibility of the catechol redox process in the pH range of 6.5–9, which could be due to the formation of a catechol–aluminum complex that is outwardly redox-inactive in the conditions of the voltammetry experiment, i.e., at this finite scan rate. At pH values higher than 9, where the capacity of HEPES may not be sufficient, the presence of aluminum restores the apparent reversibility of the redox process, but an additional, significantly more positive, anodic peak appears without a cathodic counterpart.

**Figure 3:**
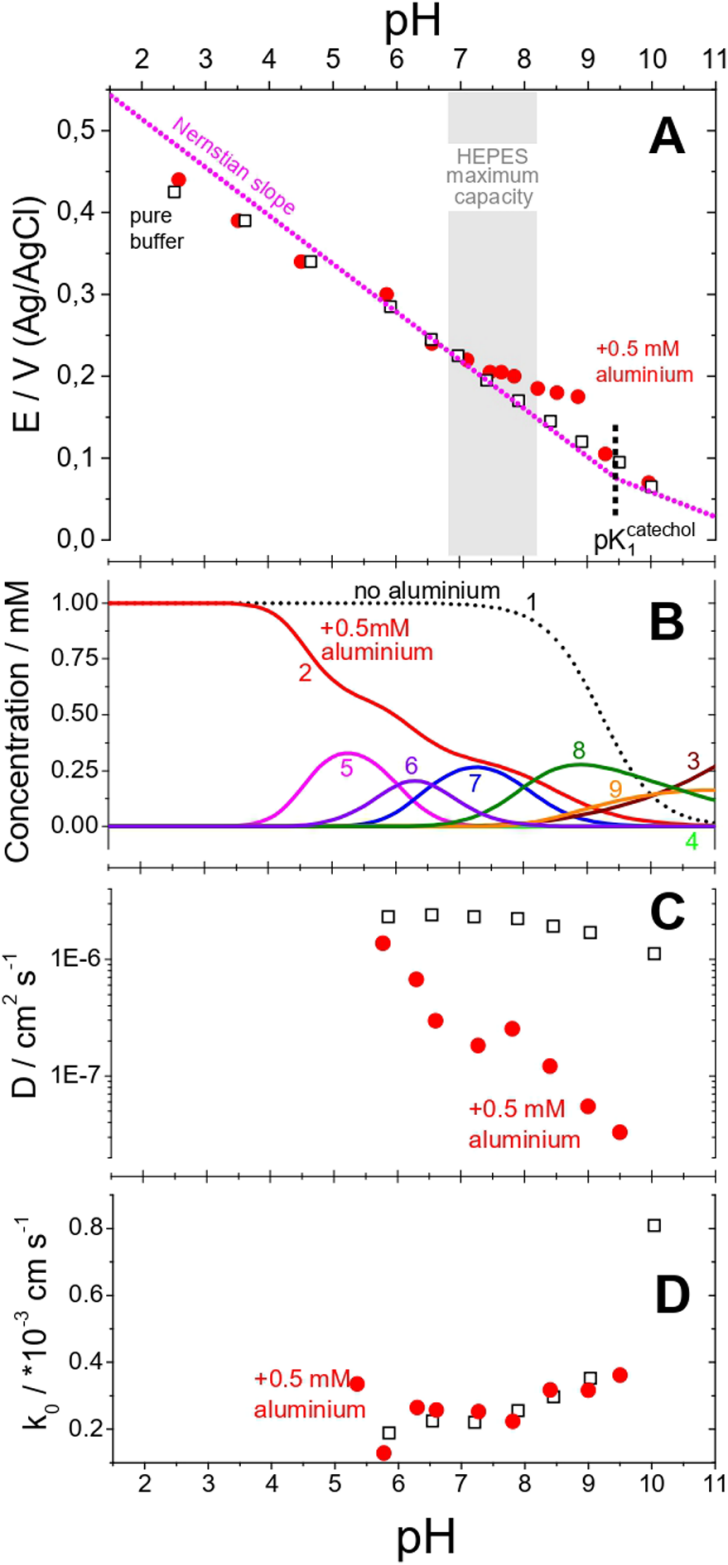
Effect of aluminum complex formation on the thermodynamics, diffusion, and kinetics of catechol oxidation. (A) Pourbaix diagram for midpoint potentials estimated by voltammetry for the catechol redox process in a pure buffer (black square) and in the presence of aluminum species (red circle); pink dashed lines: theoretical Nernstian slopes. (B) pH dependencies of the calculated equilibrium concentrations: in absence of aluminum–free catechol (dashed curve **1**); in the presence (all solid curves) of 0.5 mM aluminum–free catechol (−*CH*_2_), −anion (−*CH*^–^), and −dianion (−*C*^2–^) (curves **2**, **3**, and **4**, respectively); [*Al*(*C*^2–^)]^+^—curve **5**; *Al*(*OH*)(*C*^2–^)—curve **6**; [*Al*(*C*^2–^)_2_]^−^—curve **7**; [*Al*(*OH*)(*C*^2–^)_2_]^2–^—curve **8**; and [*Al*(*C*^2–^)_3_]^3–^—curve **9**. pH dependencies of the (C) catechol diffusion coefficient and (D) standard rate constant of catechol oxidation.

We investigated this effect on both the thermodynamics and the kinetics of the catechol redox process. A shift of the midpoint potential of the catechol redox process in the positive direction (Figures 3A and S3) illustrates the thermodynamic effect of MCET at the equilibria. Within the region of the maximum buffer capacity, the proton equilibria are intact. Therefore, the observed deviation from the Nernstian behavior is due to the aluminum species, implying the launch of secondary equilibria opposing the oxidation, which as we believe is the MCET (reaction 2) on catechol in parallel to PCET defined by the acid–base proton equilibria (reaction 2). The complexation with aluminum ions, which is the most efficient for the dianion form of catechol (*C*, Scheme 1),^20^ reduces the negative charge on the reactant, making its oxidation unfavorable.

According to analytical speciation of the aqueous aluminum–catechol system at different pH values (Figure 3B) (Supporting Note 1), these complexes of different stoichiometries are in excess over free catechol strictly in the pH range between 5 and 9. Therefore, the thermodynamic conditions of the observation of aluminum–catechol equilibria in voltammetry experiments are satisfied in this pH range. At higher pH values, aluminum complexes contain more catechols per aluminum ion, according to the speciation. pH values beyond the maximum capacity of HEPES are less buffered, which can contribute to the observed higher deviation of equilibrium of the catechol redox process from the Nernstian behavior (Figure 3A).

To resolve the kinetics of the process, we performed RDE experiments and Levich analysis of the data to explore the effect of aluminum-associated equilibria on both the diffusion (Figure 3C) and oxidation kinetics (Figure 3D) (Supporting Note 2). In the absence of aluminum, the diffusion coefficient of catechol, in the usual range of 1–2 × 10^–6^ cm^2^ s^–1^, is pH-independent. In the presence of aluminum species in equimolar concentration (1 mM Al^3+^ and catechol), the apparent diffusion coefficient of the redox-active species (i.e., calculated with the nominal catechol concentration) as a function of pH keeps decreasing consistently from the same value at a pH of around 5.5 to almost 2 orders of magnitude lower as the pH increases to 9.5. This correlates well with the onset and increase of the catechol quantity involved in the complexation equilibria with aluminum (Figure 3B). Therefore, catechol complexation suppresses the diffusion of redox species, which are now increasingly bulky complexes.

In striking contrast to cyclic voltammetry on a stagnant electrode, the presence of aluminum showed no effect on the kinetics of the catechol oxidation process (Figure 3D). Specifically, the standard (free from driving force) rate constant of the electron transfer of the catechol oxidation process is independent of the presence of aluminum in the pH region of the highest buffer capacity (Figure 3D). Lower kinetic currents and values of the heterogeneous rate constant are consistent with the formation of slower-diffusing aluminum–catechol complexes. However, at significantly higher values of overpotential, that is, driving force (Figure S6A) for the electron transfer, the standard rate constants of the electron transfer for complexed and free uncomplexed catechol were found to be similar (Figure 3D).

Two effects of complexation with aluminum on the cyclic voltammetry of the catechol redox process can be envisaged (Supporting Note 3). Redox-active catechol complexes with aluminum may diffuse more slowly to the electrode, thus decreasing peak currents at the time scale of cyclic voltammetry. Alternatively, only free uncomplexed catechol is redox-active in such experiments, while the apparent decrease in the diffusion coefficient could be due to the decrease of the redox-active material concentration.

#### 3.1.3 Unbuffered Catechol

**Figure 4:**
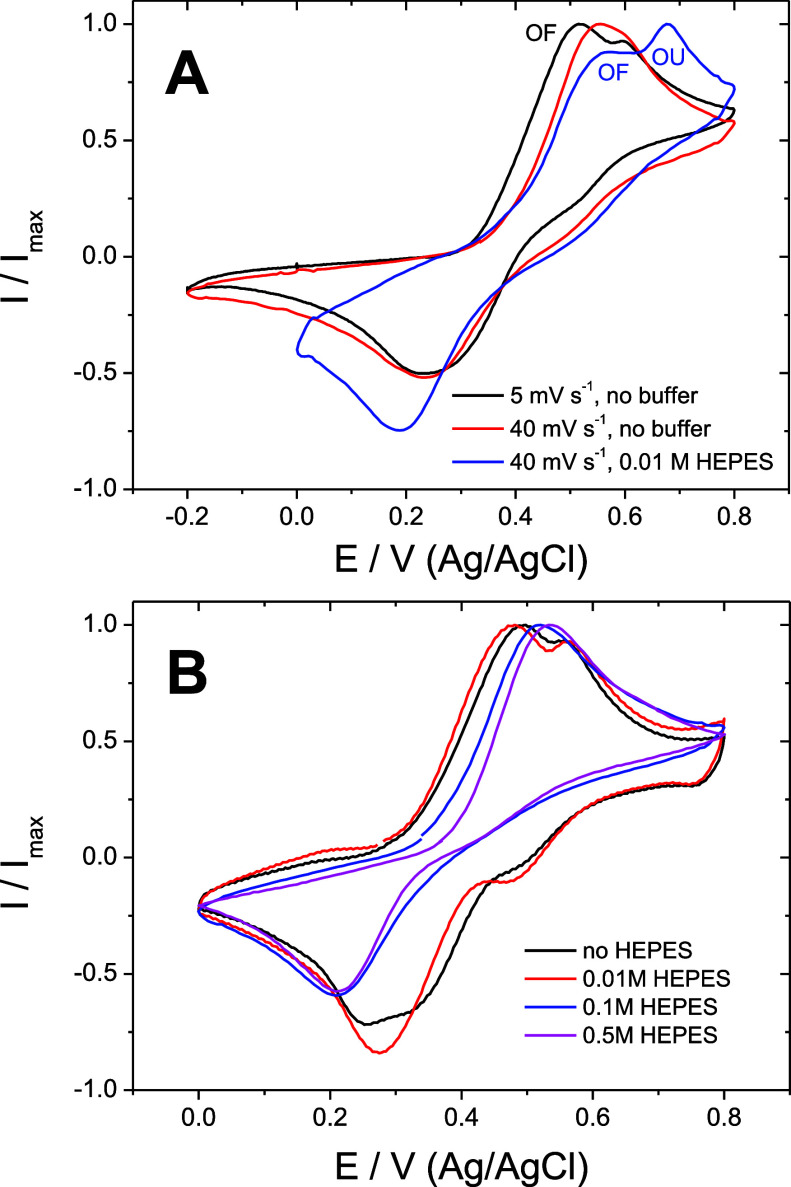
Appearance of the proton transfer limitation. (A) Voltammograms normalized by the oxidation peak of catechol (1 mM) currents acquired on a GCE at different scan rates in the absence (0.1 M KCl) and presence of the buffer (0.1 M HEPES) while the pH value was constant (pH 4.09). (B) Voltammograms normalized by the oxidation peak of catechol (1 mM in 0.1 M KCl, 20 mV s^–1^) acquired on graphite paper at different concentrations of the buffer (HEPES) while the pH value was constant (pH 4.09).

The rates of proton transfers in PCET (vertical arrows in Scheme 1) can be significantly suppressed in media of low buffer capacity. If the charge equilibration following electron transfer is assured by *slow proton transfer*, it manifests in a common voltammetry experiment on a stagnant electrode as the peak splitting into two, as demonstrated for the catechol redox process in media of low buffer capacity (Figure 4A).^8,28^ We will consider one redox process “oxidation-favorable” (OF), as it manifests at a more negative potential compared to the other, which is a more positive “oxidation-unfavorable” (OU) process. Surprisingly, an increase in the scan rate makes this split disappear, thus illustrating a far-from-equilibria behavior of the whole redox process. Furthermore, the sequential addition of buffer (increasing buffer capacity) at a constant pH (pH 4.09) showed an abrupt transition between a split peak and a single peak (Figure 4B). Importantly, the concentration of protons in the bulk of the electrolyte (bulk pH) is identical in both cases but not necessarily the local pH at the electrode where the redox process takes place. When the electrode porosity is increased by changing the working electrode from a GCE to graphite paper, the peak split is visibly restored (Figure S8). We believe that such a far-from-equilibria behavior of the catechol redox process demonstrates the conditions when the protons released upon oxidation of catechol are neutralized slowly. In other words, the rate of proton equilibration during PCET is not fast enough on the time scale of voltammetry. We believe that these kinetic considerations are important because the proton transfer sluggishness can favor MCET kinetically with respect to PCET.

## 4 Conclusions and Outlook

Our objective in this work was to investigate the impact of a redox-inactive metal ion, as a complexing and charge-compensating agent, on the observable redox processes of catechol. The aqueous medium enables the proton equilibria, which can be regulated thermodynamically by the pH and kinetically by the buffer capacity. Speciation of all sorts of aluminum complexes with catechol and buffer components was essential in understanding the electrochemical results, which incidentally stressed that the buffer can be not only a function for pH maintenance but also a true reactant. By choosing a noncomplexing buffer, in contrast to aluminum-masking media, we were able to observe complexation-driven oxidation-unfavorable MCET in the conditions of intact Nernstian PCET. The kinetic analysis of catechol oxidation at the conditions of complexation with aluminum showed significant apparent suppression of diffusion of the redox-active component, while the rate of oxidation remained intact. As the proton buffer concentration decreases, proton equilibria during catechol oxidation (PCET) are slower to establish, thus kinetically prioritizing the participation of the metal ion (MCET) instead of a proton.

For example, our experiments in buffered solutions show the absence of an aluminum ion effect at low pH, in agreement with equilibrium speciation. Nevertheless, an electrochemical signature of metal complexes is apparently observed in polycatechol films in an unbuffered medium.^29^ Our explanation would involve rapid complexation of nonequilibrium catechol dianions formed from quinone via a proton-decoupled reduction with abundant metal ions.

Understanding these aspects can be relevant in the conception of proton-free metal-ion aqueous organic batteries. Indeed, the rate of complexation to prioritize MCET can be modulated by chelating agents. Therefore, a general strategy to achieve selectivity on MCET relevant for organic battery cathodes^18^ would include

- a low proton buffer capacity,
- a high concentration of metal-specific chelating agent in free and metal-loaded forms,
- the absence of any general cations except the potential-determining metal ion, and
- electrodes of high porosity.

However, the kinetic volatility controlled by at least five parameters, namely, the scan rate (or charge–discharge current density), electrode porosity, electron-transfer kinetics, buffer capacity (proton transfer rate), and finally the rate of complex formation with metal ions, can become a significant challenge for such technologies.

To conclude, we believe that this investigation of the effect of the aluminum ion complexation on the catechol redox process is relevant for both bioadhesion and electrical energy storage in organic materials.

## Supporting Information Available

The Supporting Information contains a figure illustrating the cyclic voltammetry of catechol in the borate-free Britton–Robinson buffer, Supporting Note 1 on the speciation of catechol species in the absence and presence of aluminum, the Pourbaix diagram for the calculated midpoint potentials for the pure buffer, Supporting Note 2 on the kinetics of catechol oxidation, a figure illustrating the confinement of the standard rate constant of the electron transfer of catechol oxidation in the presence of aluminum, Supporting Note 3 on the modeling of the complexation effect on the voltammetry of the catechol redox process, Table S1 on the compositions of the borate-free Britton–Robinson buffers, and a figure illustrating the effect of the electrode surface area on the proton transfer limitation (PDF)
