# Supplementary material for: Metal Ion-/Proton-Coupled Electron Transfer (MPCET) on ortho-Quinone
Source: ACS Omega. 2024 Sep 5;9(37):38498–505. doi: 10.1021/acsomega.4c03621 (PMC11411522; doi:10.1021/acsomega.4c03621)
Supplement: Supplementary file 1 — ao4c03621_si_001.pdf [file ao4c03621_si_001.pdf]

Supporting Information

Metal ion-/proton-coupled electron transfer (MPCET) on *ortho*-quinone

Divyaratan Kumar,[1,2] Viktor Gueskine,[1,2] Ziyauddin Khan,[1] Reverant Crispin,[1,2,3]
Mikhail Vagin[1,3]*

[1]Laboratory of Organic Electronics, Department of Science and Technology, Linköping University, SE-60174 Norrköping, Sweden
[2]Wallenberg Wood Science Center, ITN, Linköping University, Norrköping, Sweden
[3]Wallenberg Initiative Materials Science for Sustainability, Department of Science and Technology, Linköping University, Norrköping 60174, Sweden

*Corresponding author: mikhail.vagin@liu.se, +46702753087

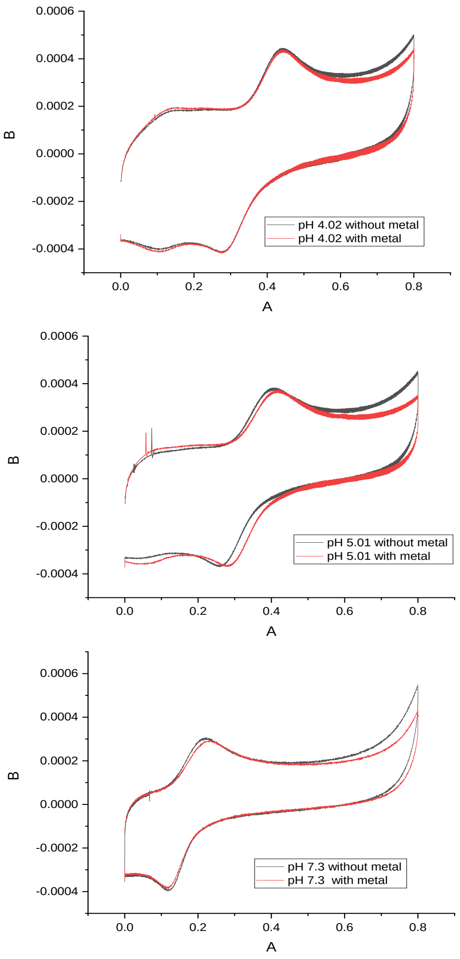

Figure 1S. The cyclic voltammetry of catechol (0.02 mM) in borate-free Britton-Robinson buffer; scan rate 20 mV s^{-1}.

Supporting Note 1.

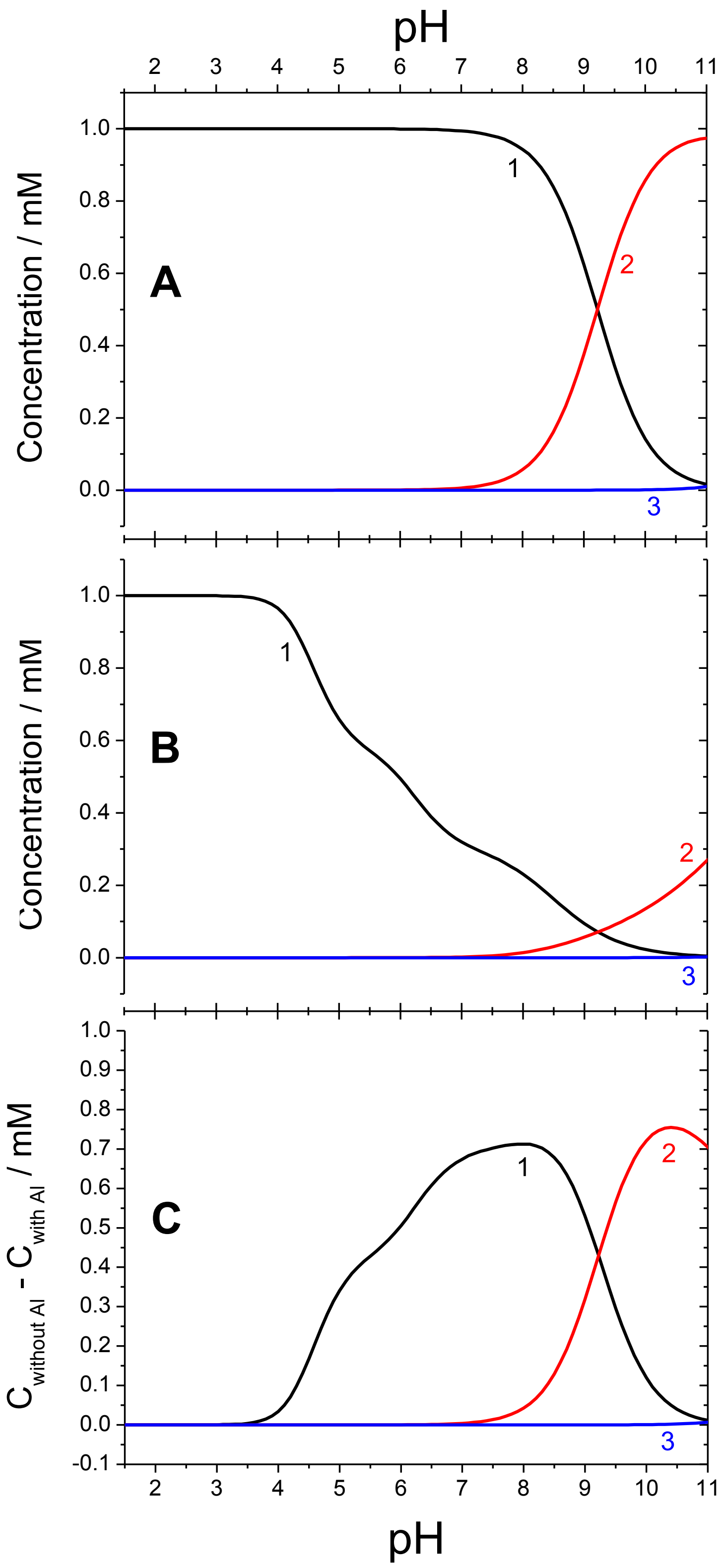

Figure 2S. pH dependencies of calculated equilibrium concentrations for catechol species. In absence (**A**) and in presence (**B**) of aluminum (0.5 mM); **C:** the difference between equilibrium

concentrations estimated without and with aluminium; free catechol (CH_2), its anion (CH^-) and di-anion (C^{2-}, curves **1**, **2** and **3**, respectively),

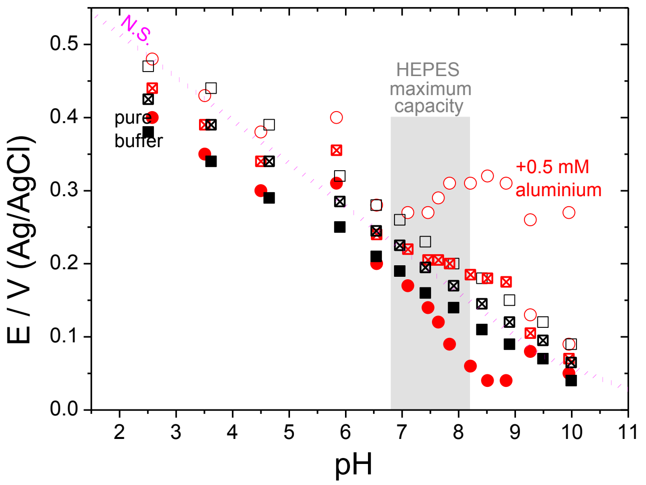

Figure 3S. Pourbaix diagram for voltammetry peak currents and calculated mid-point potentials for pure buffer (black symbols) and in presence of aluminium species (red symbols; ● and ■ - potentials of reduction peak currents, ○ and □ - potentials of oxidation peak currents, ☒ and ☒ - mid-point potentials).

Supporting Note 2.

Kinetics study of catechol oxidation by RDE voltammetry

The launching of disk electrode rotation during the voltammetry measurements led to the enhancement of the diffusion of catechol from the bulk of electrolyte to the electrode and the product of oxidation, namely *o*-benzoquinone, in opposite direction. This led to the typical transformation of the voltametric response (Fig. 4SA) from the curve featured with peak current obtained on stagnant electrode to the s-shaped curve featured with a region of potential-independent currents, so-called limiting currents.

The dependence of the limiting current on the angular frequency of rotation is Levich equation:

$$I_{lim} = 0.62nFAD^{2/3}\upsilon^{-1/6}\omega^{1/2}C \qquad (1)$$

where I_{lim} is the limiting current of catechol oxidation (A), n is the number of transferred electrons (for quinones $n = 2$), F is the Faraday constant (96485 C mol^{-1}), A is the geometrical area of RDE (cm^2), D is the diffusion coefficient of catechol (cm^2 s^{-1}), υ is the kinematic viscosity of solvent (0.009 cm^2 s^{-1} for water at 25 ^{o}C), ω is the angular frequency of rotation (rad s^{-1}, which is calculated as $\omega = \frac{2\pi q}{60}$, where q is the rotation speed of RDE (rpm)) and C is the catechol concentration (1×10^{-6} mol cm^{-3} for 1 mM solution). The diffusion coefficient of catechol can be estimated from the slope of the linear dependence of the limiting current vs $\omega^{1/2}$ (Fig. 4SB).

In parallel, the control of diffusion by means of hydrodynamics on RDE enables the subtraction of diffusional contribution from the total recorded current yielding the kinetic diffusion-free current defined by heterogeneous electron transfer only. The estimation of kinetic current was done by analysis of voltammograms obtained at the different rotation speeds using Koutecky-Levich equation imposed on the electrode reaction under kinetic control:

$$\frac{1}{I} = \frac{1}{I_K} + \frac{1}{I_L} = \frac{1}{I_K} + \frac{1}{B\omega^{1/2}} \qquad (2)$$

where I is the total recorded current on RDE, I_K is the kinetic current free from any diffusion limitations, B is a constant. The plot of the reciprocal total current $\left(\frac{1}{I}\right)$ against the reciprocal root of the angular rotation rate $\left(\omega^{-1/2}\right)$ (Koutecky-Levich plot, Fig. 4SC) gives a straight line, which verifies the assumption of kinetic control. In this case, the intercept with the vertical axis is equal to the reciprocal kinetic current $\left(\frac{1}{I_K}\right)$. The kinetic current depends linearly on the concentration:

$$I_K = nFAk_HC \qquad (3)$$

where k_H is the heterogeneous rate constant for electron transfer (cm s^{-1}). The driving force of oxidation, so-called overpotential (η, V), is the difference between the applied potential E and the mid-point potential E_0 estimated from cyclic voltammetry on stagnant electrode. Due to its exponential character, the dependence of kinetic current on the overpotential can be presented in semi-logarithmic coordinates ($ln(I_K)$ vs η), so-called Tafel plot (Fig. 4SD), where the linearization slope is reciprocal to Tafel slope, the inherent kinetic parameter characterizing the mechanism of electrode reaction.

The heterogeneous rate constant depends on the driving force, namely overpotential, of the electrode process:

$$k_H = k^{0\prime} e^{\frac{(1-\alpha)F}{RT}\eta} \qquad (4)$$

where $k^{0\prime}$ is the standard (overpotential-independent) rate constant of the electron transfer (cm s^{-1}), α is the transfer coefficient ($0 < \alpha < 1$), R is the gas constant (8.31 J K^{-1} mol^{-1}) and T is a temperature (298 ^{o}K). The logarithm of (4) gives:

$$ln(k_H) = ln(k^{0\prime}) + \frac{(1-\alpha)F}{RT}\eta \qquad (5)$$

The plot of $ln\left(k_{H(oxidation)}\right)$ against overpotential η (Fig. 5SA) at the conditions of the independence of the slope of Koutecky-Levich plot on the potential (Fig. 5SB) [S. Treimer, A. Tang, D.C. Johnson. Electroanalysis, 2002, 14(3), 165-171] gives the straight line. Its slope and intercept allow to estimate the transfer coefficient and the standard rate constant of electron transfer, respectively.

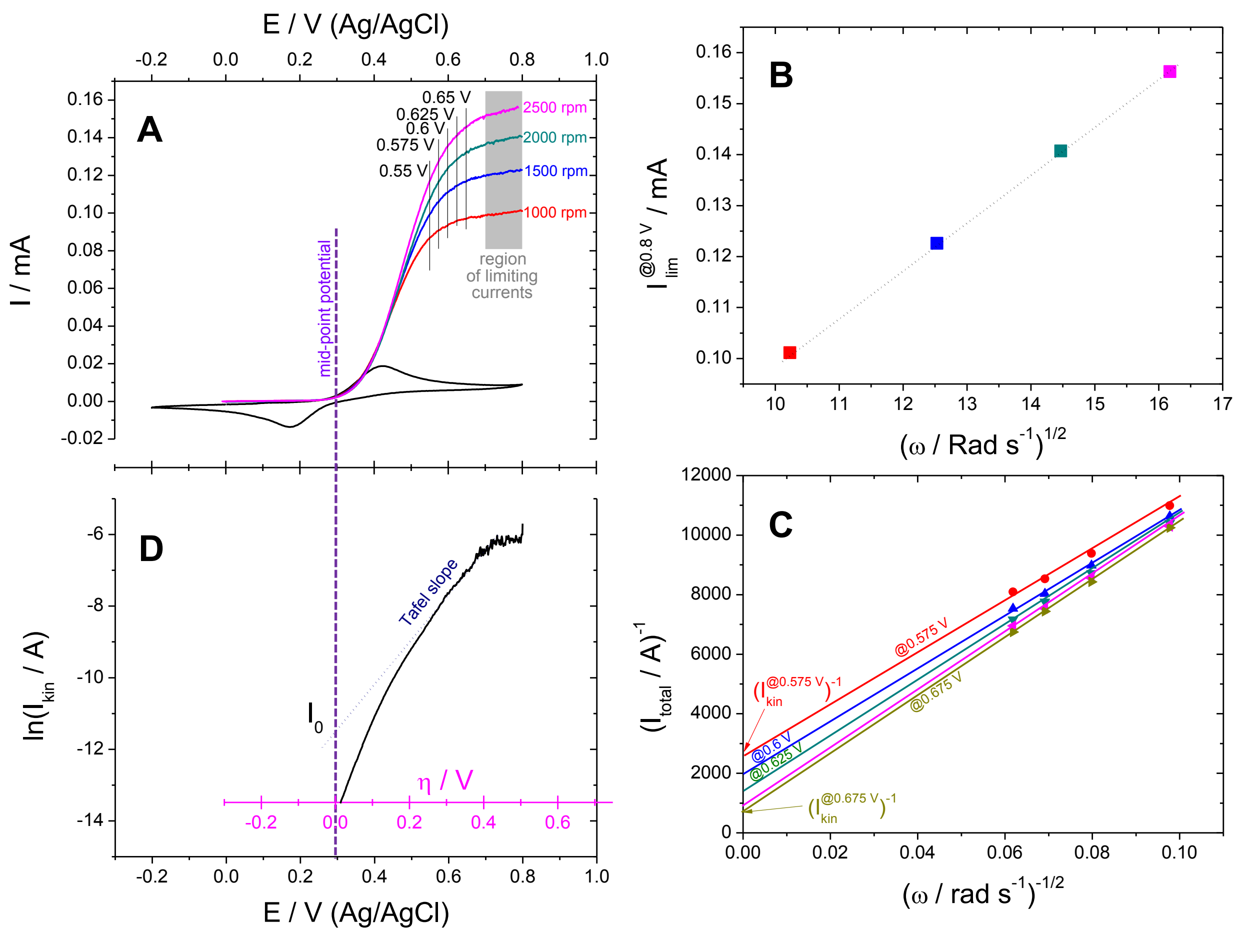

Figure 4S. A Levich study of catechol oxidation in absence of aluminium. **A:** the RDE voltammetry in catechol solution (1 mM in HEPES 1M, 0.1M KCl, pH 5.86) in absence of aluminium (black curve – cyclic voltammetry on stagnant electrode, colored curves – linear sweep voltammograms on RDE at different rotation rates; scan rate 20 mV s^{-1}); **B:** the dependence of the limiting current (at 0.8 V) on the square root of the angular frequency; **C:** Koutecky-Levich plot of voltammetry data of Fig. 4SA; **D:** Tafel plot.

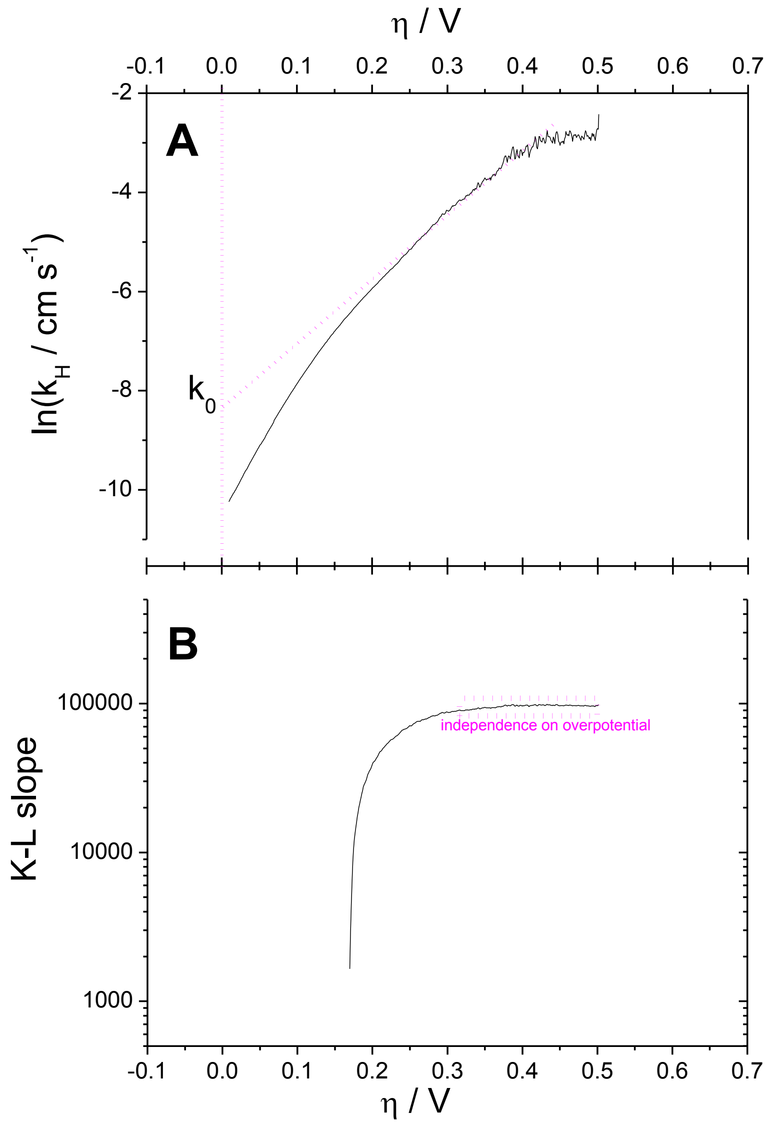

Figure 5S. The estimation of the standard rate constant of the electron transfer from Koutecky-Levich plot. The dependences of the logarithm of heterogeneous rate constant depends (**A**) and the slope of Koutecky-Levich plot (**B**) on the overpotential η.

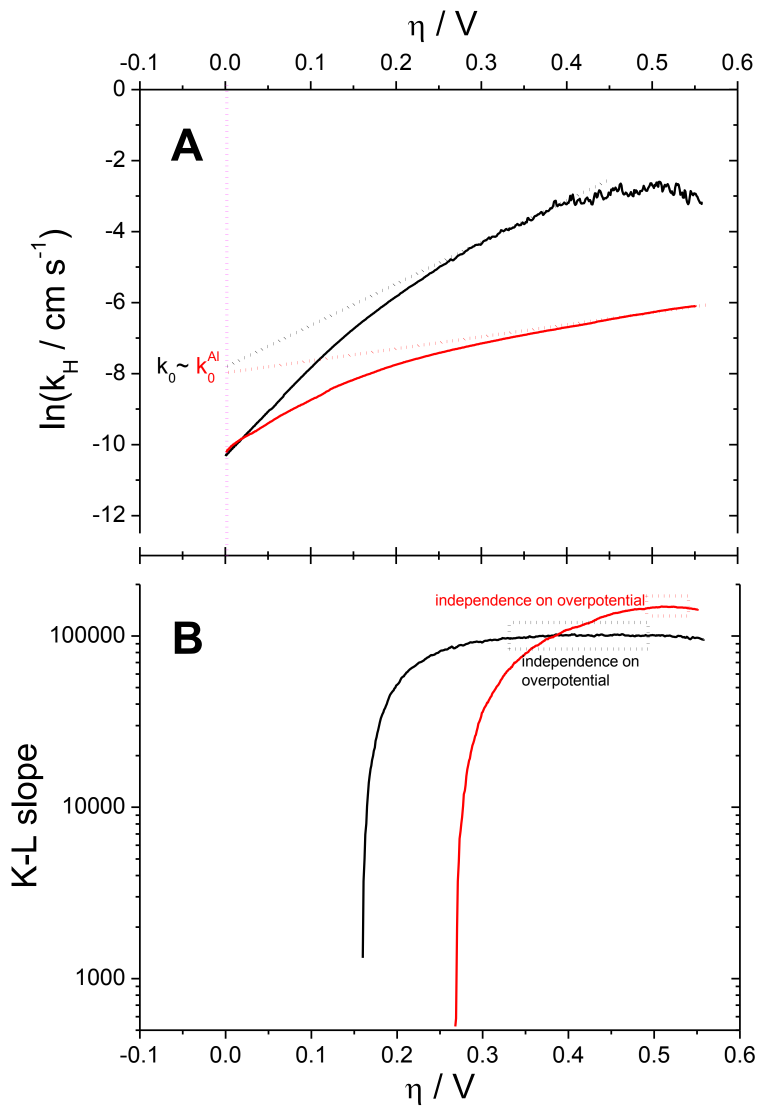

Figure 6S. The intactness of the standard rate constant of the electron transfer of catechol oxidation in the presence of aluminium. The dependences of the logarithm of heterogeneous rate constant depends (**A**) and the slope of Koutecky-Levich plot (**B**) on the overpotential η obtained in absence and in presence of aluminium (black and red curves, respectively), pH 7.27 HEPES.

Supporting Note 3.

Numeric modeling of aluminum complexation effect on cyclic voltammetry of catechol redox process

We performed numeric simulation of cyclic voltammograms (CV) obtained for catechol redox process in non-masking buffer HEPES (Fig. 2) using the computational approach developed by Q. Lin et al. [15]. Specifically, the CV of catechol was represented individually at each pH value by two effectively apparent non-proton coupled single-electron transfers:

$$Q + e^- \leftrightarrow SH \quad (6)$$

with apparent standard potential $E^0_{1,apparent}$ and

$$SH + e^- \leftrightarrow CH_2 \quad (7)$$

with apparent standard potential $E^0_{2,apparent}$.

The rate constants for both (6) and (7) were set to 10 cm s^{-1}, while diffusion coefficient of catechol was set to 1.1×10^{-5} cm^2 s^{-1}. The values of apparent standard potentials estimated for pH values from 1 to 5 follows the linearity:

$$E^0_{1,apparent} = 0.0143 - 0.0464 \times pH \quad (8)$$

$$E^0_{2,apparent} = 1.040 - 0.0570 \times pH \quad (9)$$

This implies that the whole system shows the voltametric behavior phenomenologically equal to PCET.

We extended the pH range to 8 using (8) and (9) in order to enter the pH range of aluminum complexation to catechol (Figure 3B), while avoiding first de-protonation of catechol at $pK_{a1}^{catechol} = 9.45$ and thus still in the pH domain of reactions (6) and (7).

1. In the first model, we assume that catechol moiety remains outwardly redox active in all complexes with aluminum at the conditions of voltammetry experiment with a certain finite scan rate. Therefore, we used the total catechol concentration (1mM). In parallel, the complexes with aluminum are charged and bulky, unlike free catechol, they diffuse slower leading to the observable diffusion coefficient decrease. Therefore, we used the values of apparent diffusion coefficient D from Levich analysis of RDE data (Fig. 3C). The result (Fig. 7SA) shows notable decrease of redox peak currents at the certain CV time scale with increasing pH, as more redox active but slower complexes are formed.

2. In the second model, we assume that only free un-complexed catechol remains redox-active. The complexation with aluminum yields outwardly redox-inactive catechol in all complexes with aluminum at the conditions of voltammetry experiment. The electrode reaction mechanism based on both (6) and (7) was updated with the series of aluminum-associated chemical equilibria:

$Al + CH_2 \leftrightarrow AlC$

$AlC + CH_2 \leftrightarrow AlC_2$

$AlC_2 + CH_2 \leftrightarrow AlC_3$

$Al + CH_2 \leftrightarrow Al(OH)C$

$Al(OH)C + CH_2 \leftrightarrow Al(OH)C_2$

with the complexation constant values used in theoretical calculations of equilibrium concentrations.

The result (Fig. 7SB) shows gradual decrease of oxidation peak currents with increase of pH as soon as the concentration of free un-complexed catechol decreases due to the conversion to redox-inactive complex with aluminum. Importantly, such an explanation of peak current decrease due to the formation of redox-inactive complexes does not contradict the estimation of apparent diffusion coefficient by Levich equation ((1) and Fig. 3C). The slope of the linear dependence of the limiting current vs $\omega^{1/2}$ (namely, $slope = 0.62nFA\upsilon^{1/6}CD^{2/3}$), is contributed by both bulk concentration of the redox active substance C and the apparent diffusion coefficient entering as $D^{2/3}$. Apparent D was calculated using Levich equation on the assumption of constant total concentration of catechol, so all the changes were assigned to decreasing D. However, decreasing of concentration of redox active catechol at constant $D^{2/3}$is also a formally coherent interpretation. Furthermore, roughly two-orders of magnitude decrease of apparent D, that is 20-fold decrease of $D^{2/3}$, is compatible with the evolution of concentration of free catechol (curve 2 in Fig. 3B).

At this point, we lack data to choose between these two models.

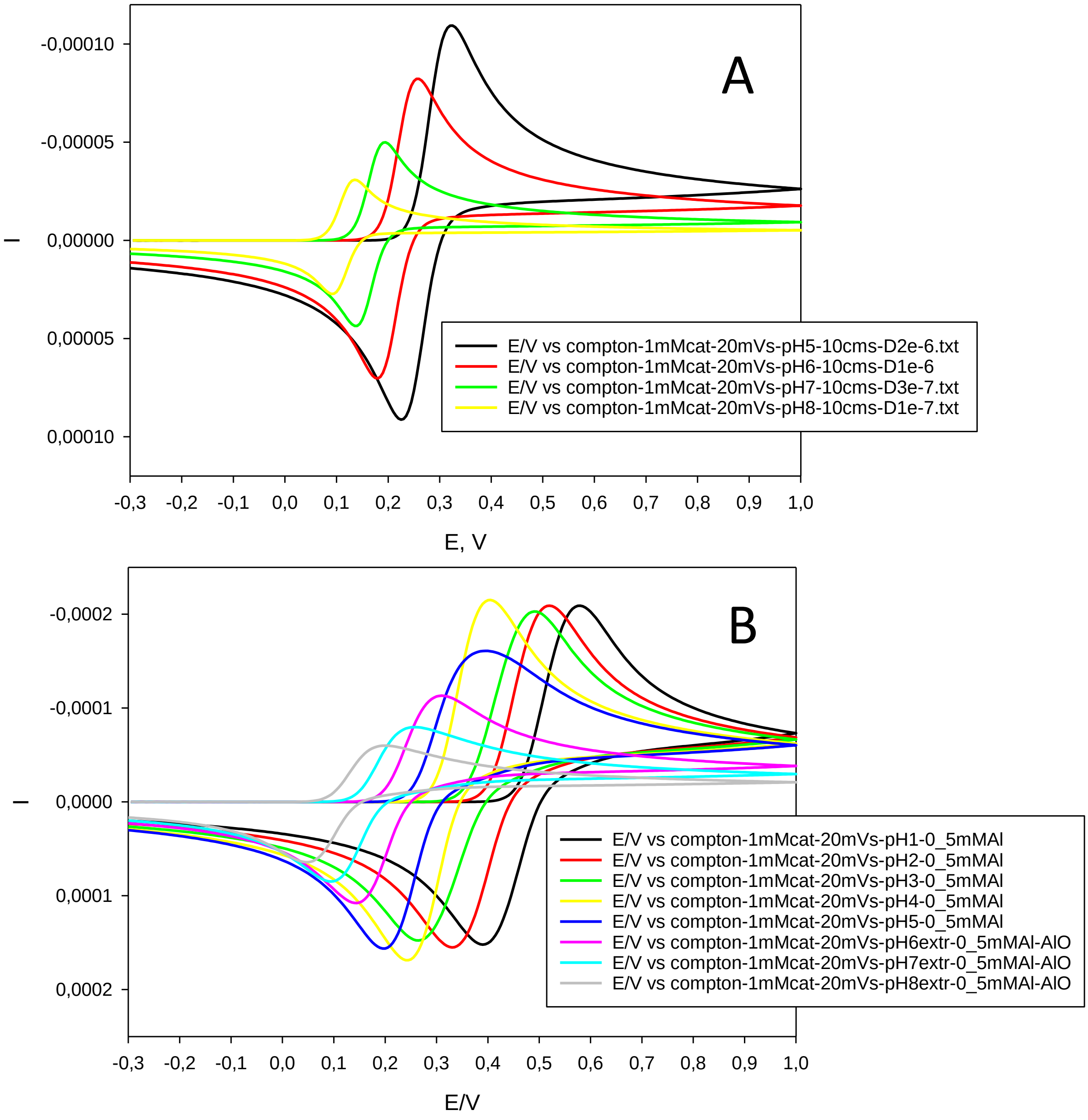

Figure 7S. Simulated cyclic voltammograms (scan rate 20 mV/s) for redox process of catechol (1 mM) at different pH of non-complexing buffer (HEPES). **A:** the effect of the suppression of apparent diffusion coefficient estimated from Levich study; **B:** the effect of catechol conversion to the redox-inactive complex with aluminum (0.5mM Al^{3+}).

Table 1S. The compositions of the borate-free Britton-Robinson buffers.

pH interval	Concentration, g L^{-1}				Molar concentration, M				Total concentration, M
	NaOH	CH_3COOH	H_3PO4	KCl	NaOH	CH_3COOH	H_3PO4	KCl	
2.1	0.558	2.234	3.647	5.964	0.014	0.037	0.037	0.08	0.168
2.9	1.191	2.044	3.336	5.203	0.03	0.034	0.034	0.07	0.168
4.1	1.6	1.922	3.136	4.562	0.04	0.032	0.032	0.06	0.164
5.0	2.074	1.779	2.904	3.683	0.052	0.03	0.03	0.05	0.162
6.1	2.386	1.686	2.751	2.959	0.06	0.03	0.028	0.04	0.158
7	2.754	1.575	2.57	1.804	0.07	0.026	0.026	0.024	0.146
8	3	1.501	2.45	0.357	0.075	0.025	0.025	0.005	0.13
9	3.224	1.434	2.34	0	0.081	0.024	0.024	0	0.129
9.9	3.493	1.353	2.208	0	0.087	0.023	0.023	0	0.133
10.9	3.616	1.316	2.148	0	0.09	0.022	0.022	0	0.134
12	4	1.201	1.96	0	0.1	0.02	0.02	0	0.14

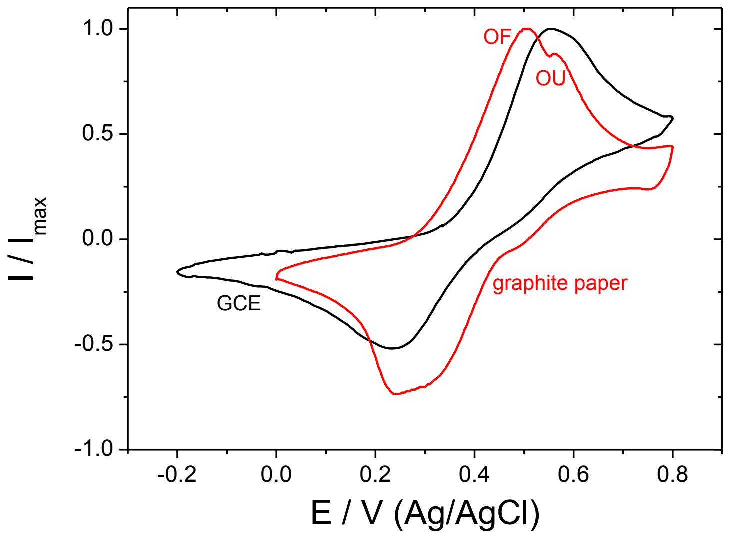

Figure 8S. The influence of the electrode surface area on the proton transfer limitation. The voltammograms normalized by the oxidation peak of catechol (1 mM) acquired on GCE and graphite paper (40 mV s^{-1}, 0.1M KCl, no buffer).
